# Toll-Like Receptor 4–Mediated Nuclear Factor Kappa B Activation Is Essential for Sensing Exogenous Oxidants to Propagate and Maintain Oxidative/Nitrosative Cellular Stress

**DOI:** 10.1371/journal.pone.0073840

**Published:** 2013-09-18

**Authors:** Rajendra Karki, Orisa J. Igwe

**Affiliations:** Division of Pharmacology and Toxicology, University of Missouri-Kansas City, Missouri, United States of America; UAE University, Faculty of Medicine & Health Sciences, United Arab Emirates

## Abstract

The mechanism(s) by which cells can sense exogenous oxidants that may contribute to intracellular oxidative/nitrosative stress is not clear. The objective of this study was to determine how cells might respond to exogenous oxidants to potentially initiate, propagate and/or maintain inflammation associated with many human diseases through NF-κB activation. First, we used HEK-Blue cells that are stably transfected with mouse toll-like receptor 4 (mTLR4) or mouse TLR2. These cells also express optimized secreted embryonic alkaline phosphatase (SEAP) reporter gene under the control of a promoter inducible by NF-κB transcription factor. These cells were challenged with their respective receptor-specific ligands, different pro-oxidants and/or inhibitors that act at different levels of the receptor signaling pathways. A neutralizing antibody directed against TLR4 inhibited responses to both TLR4-specific agonist and a prooxidant, which confirmed that both agents act through TLR4. We used the level of SEAP released into the culture media due to NF-κB activation as a measure of TLR4 or TLR2 stimulation. Pro-oxidants evoked increased release of SEAP from HEK-Blue mTLR4 cells at a much lower concentration compared with release from the HEK-Blue mTLR2 cells. Specific TLR4 signaling pathway inhibitors and oxidant scavengers (anti-oxidants) significantly attenuated oxidant-induced SEAP release by TLR4 stimulation. Furthermore, a novel pro-oxidant that decays to produce the same reactants as activated phagocytes induced inflammatory pain responses in the mouse orofacial region with increased TLR4 expression, and IL-1β and TNFα tissue levels. EUK-134, a synthetic serum-stable scavenger of oxidative species decreased these effects. Our data provide *in vitro* and related *in vivo* evidence that exogenous oxidants can induce and maintain inflammation by acting mainly through a TLR4-dependent pathway, with implications in many chronic human ailments.

## Introduction

Oxidative/nitrosative stress (ONS) induced by reactive oxygen species (ROS) and reactive nitrogen species (RNS) are said to be an important initiating factor in many human diseases with little or no effective treatment [1]. ONS may be caused by an imbalance in the generation and removal of ROS/RNS [2]. These oxidative species are implicated in signal transduction and gene activation that may play a role in initiating, propagating and maintaining several disease states [3], [4]. It is well established that oxidants are involved in cellular signaling, cell growth, and inflammation [5], [6].

Substantial amounts of ROS (used in this manuscript to also encompass RNS) are generated from endogenous (internal) sources as by-products of normal and essential metabolic reactions. It is not clear whether and how exogenous (external) oxidants may play a role in regulating the levels of endogenous oxidants, thereby increasing cellular ONS that contributes to the propagation and maintenance of different disease states. Nonetheless, exogenous sources of oxidants that may impact on the levels of endogenous oxidants include exposure to cigarette smoke, environmental pollutants, radiation and infectious agents [7], [8]. There is still considerable ongoing debate about how cells can sense oxidants and how they may propagate the inflammatory response. Therefore, it is important to understand the mechanism(s) involved in cellular oxidant sensing because of the role of ONS in many life-threatening diseases [9] including chronic pain [10].

NF-κB, a transcription factor that regulates the expression of many genes involved in immune and inflammatory response, is considered to be oxidant-responsive [11]. However, the mechanism(s) by which oxidants regulate NF-κB activation has remained elusive. Many reports have documented the role of oxidative stress in NF-κB translocation by various inflammatory stimuli including lipopolysaccharide (LPS) [12]. Inflammation induced by oxidant stress has many of the features associated with classical activation of the innate immune system and, as such, resemble that seen after activation of toll-like receptors (TLRs) with LPS.

TLRs are evolutionarily conserved type I membrane glycoproteins that recognize molecular structures shared by a wide range of pathogens known as pathogen associated molecular patterns (PAMPs) [13]. In addition, TLRs can also respond to endogenous molecules released in response to stress, trauma, and cell damage, which are collectively known as damage associated molecular patterns (DAMPs) including non-host non-pathogenic environmental factors [14]. TLRs are predominantly expressed in immune cells including polymorphonuclear leukocytes (PMNs), macrophages, microglia and dendritic cells as well as on certain non-immune cells such as endothelial and muscle cells [15]. Upon activation by PAMPs or DAMPs, TLRs can then induce the recruitment of different adaptor proteins [16] to regulate their biological functions.

The emergence of a new role for non-pathogenic-associated sensing by TLRs has increased their biologic repertoire, such that TLRs, especially TLR4 and TLR2, may now also be considered “general” surveillance receptors for danger signals [17], [18]. It has been shown that constitutively active TLR4 can induce the activation of NF-κB with consequent expression of a number of proinflammatory cytokines and a co-stimulatory molecule [19]. TLR4 has been speculated as a potential therapeutic target in neuropathic and other chronic pain states. Therefore, it is important to determine how TLR4 activation may be regulated not only at the receptor expression level, but also through its signaling pathway. Understanding the mechanism of an integrated TLR4 functions has the potential to provide us with new opportunities for developing new therapeutic agents for use in chronic diseases [20] including chronic pain states [21], [22], [23]. Orofacial pain encompasses a range of debilitating conditions [24], [25]. Recent studies have demonstrated that the TLR4 is expressed in the capsaicin receptor and the vanilloid receptor 1 (TRPV1)-containing trigeminal sensory neurons [26] that supply touch–pain–temperature sensations to the face. Given the prevalence of orofacial pain and its economic cost, studies that would provide insights into TLR4 signaling in the orofacial region would have the potential to open a new avenue for the management of this difficult-to-treat clinical pain.

The potential for exogenous oxidants to interact with the signaling pathway of PAMPs and DAMPs in health and disease remains undefined. The purpose of this study was to determine to what extent exogenous pro-oxidants activate NF-κB through TLR4 and/or TLR2. First, with cellular model systems that express either TLR4 with the CD14/MD-2 co-receptor genes, or TLR2 with the CD14 co-receptor gene against a null background, we examined the role of pro-oxidants on TLR4- and TLR2- dependent NF-κB activation. To integrate the *in vitro* system with an *in vivo* model, we used an orofacial behavioral pain model. We investigated the role of a novel prooxidant in regulating the expression of TLR4 and the levels of proinflammatory cytokines in the mouse masseter (masticatory) muscle.

## Experimental Procedures

### Chemicals

HEK-Blue™ selection medium, selection antibiotic Zeocin, Quanti-Blue detection reagent (alkaline phosphatase detection medium), synthetic monophosphoryl lipid A (MPLA), LPS from *Rhodobacter sphaeroides* (LPS-RS), LPS from *Porphyromonas gingivalis* (LPS-PG), CLI-095, BAY11-7082 and polyclonal rat IgG used as pAb neutralization control, were obtained from InvivoGen (San Diego, CA). Potassium dioxide (PDO) and dimethyl sulfoxide (DMSO) were obtained from Sigma (St. Louis, MO), whereas linsidomine chloride (SIN-1) and sodium peroxynitrite (PN) were obtained from Cedarlane Inc (Burlington, NC). N-Tert-butyl-α-phenylnitrone (PBN) and 18-crown-6-ether were from Acros Organics (Fairlawn, NJ), while Ebselen was from Enzo Life Sciences (Farmingdale, NY). Rabbit anti-nitrotyrosine and nitrotyrosinated bovine serum albumin were purchased from Cell Biolabs (San Diego, CA); toll-interleukin 1 receptor (TIR) adaptor peptide (TIRAP**_138-151_**) fused to the *Drosophilia antennapedia* inhibitor and its scrambled non-functional sequence TIRAP control (TIRAPc) were obtained from EMD Millipore (Bellerica, MA). Low endotoxin, azide-free (LEAF) affinity purified rat IgG2a, κ-isotype anti-mouse TLR4 (CD284)/MD-2 complex pAb for TLR4 neutralization was purchased from Biolegend (San Diego, CA). EUK-134 was purchased from Cayman Chemical (Ann Arbor, MI) and anti-TLR4 IgG pAb was from Abcam (Cambridge, MA). Ninety-six well microplate kits for sandwich ELISA of mouse-specific TNF-α and IL-1β were purchased from Thermo Fisher Scientific/Pierce (Rockford, IL).

### Preparation of Potassium Peroxychromate (PPC)

Because PPC is not commercially available, it was prepared in the laboratory according to a published protocol [27]. The product was characterized by elemental and infrared analyses, and the purity was determined to be >98%.

### Cell Lines and Culture

Cells were derived from the human embryonic kidney-293 (HEK-293) cell line. HEK-Blue-Null1-v, HEK-Blue mouse TLR2 (mTLR2) and HEK-Blue mTLR4 cells were purchased from InvivoGen (San Diego, CA). HEK-Blue Null1-v is the parental cell line of HEK-Blue mTLR4, but does not express mTLR2 or mTLR4. HEK-Blue mTLR2 cells are stably transfected with mTLR2 gene with the co-receptor CD14. Similarly, HEK-Blue mTLR4 cells are stably transfected to express mTLR4 gene at high levels with MD-2 and CD14 co-receptor genes involved in TLR4 recognition and presentation. In addition, these cells stably express an optimized secreted alkaline phosphatase (SEAP) reporter gene under the control of a promoter inducible by NF-κB and activator protein-1 (AP-1) transcription factors. HEK-Blue™ Null v1 cells also express the SEAP reporter gene under the control of the IFN-β minimal promoter fused to NF-κB and AP-1 binding sites. The level of SEAP protein released into the culture media was used to quantify the extent of TLR2 or TLR4 stimulation, which also represents the levels of NF-κB activation.

Cells were grown in a 37°C, 100% humidified incubator in Dulbecco’s Modified Eagle’s Medium (DMEM, 4.5 g of glucose/L) without pyruvate but supplemented with 2 mM L-glutamine, 10% (v/v) fetal bovine serum (FBS), 50 units/ml penicillin, 50 µg/ml streptomycin and 100 µg/ml Normocin™. HEK-Blue mTLR2 and mTLR4 were maintained in growth medium supplemented with HEK-Blue selection reagent.

### Cell Viability Assay

HEK-Blue-Null1-v, HEK-Blue mTLR2 or HEK-Blue mTLR4 cells were plated at a density of 5×10^5^ cells/well. Cells were grown to 70% confluence on 96-well plates in culture medium consisting of DMEM with 10% FBS and antibiotics. To induce oxidative conditions, cells were incubated overnight in culture medium containing varying concentrations of PPC, PDO or PN. Cells were then labeled with (3-(4,5-dimethylthiazol-2-yl)-2,5-diphenyltetrazolium bromide (MTT) for 2 h followed by dissolution of the violet crystals in DMSO. Finally, we assessed the cell viability by measuring the absorbance at 570 nm in a Bio-Tek® microplate reader (Burlington, VT).

### Quanti-Blue SEAP Reporter Assay

HEK-Blue-Null1-v, HEK-Blue mTLR2 or HEK-Blue mTLR4 cells were plated in 96-well plates and grown to 70% confluence. Cells were then treated with various inhibitors for 30 min followed by stimulation with MPLA, LPS-PG, PPC, PDO or PN for 16 h. Then, 20 µl aliquots of cell culture medium were removed and added to plates containing 180 µl of pre-warmed Quanti-Blue detection reagent per well as per manufacturer’s instructions. Color was allowed to develop for 1 h, and absorbance was read at 650 nm in Bio-Tek® microplate reader (Burlington, VT).

### TLR4 Neutralization Assay

To ensure that we were indeed working with mTLR4, we neutralized the receptor with an anti-mouse TLR4 raised in rat. The cells were preincubated with anti-mouse TLR4 (5 µg/ml) for 2 h before stimulation over night with MPLA (57 nM) or PPC (1 µM) in continued presence of the neutralizing antibody. We used polyclonal rat IgG (10 µg/ml) as a neutralization control. The level of SEAP released into the culture media was used to quantify the extent of TLR4 stimulation/inhibition.

### Immuno-blotting Assay

HEK-Blue mTLR2 and HEK-Blue mTLR4 cells were plated in 6-well plates at a density of 5×10^5^ cells/plate. Cells were allowed to reach 70% confluence and then treated with equimolar concentration (1 mM) of PN or SIN-1 for 3 h to compare parallel tyrosine nitration efficiencies of these agents under the same conditions. Cells were then lysed in mammalian cell protein extraction lysis buffer (Mammalian cell-PE-LB™) (G Biosciences, St. Louis, MO) supplemented with protease inhibitors. Cell lysates were centrifuged at 16,200-×g at 4°C for 10 min. Aliquots of cell extracts containing 30 µg of total protein were mixed with the loading buffer in a total volume of 30 µl, and heated at 90°C. Equal amounts of the denatured protein (contained in 15 µl) were loaded per lane, fractionated on a 4–12% Bis-TRIS electrophoresis gel, and transferred onto polyvinylidene diflouride (PVDF) membranes. After blocking membranes with 5% nonfat dry milk in TRIS buffered saline with 0.1% Tween-20 (TBS-T), membranes were incubated overnight with rabbit anti-nitrotyrosine (1∶1000). Following three washes in TBS-T, membranes were incubated for 1 h with anti-rabbit IgG (1∶5000) secondary antibody. After additional washes, membranes were developed by ECL chemiluminescent method and signals were visualized with the Fujifilm LAS-400 imaging system.

### Animals and Treatments

Pathogen-free male JAX® strain C57BL/6J mice (Stock #:000664) weighing (20–25 g; 10–12 weeks old) were used as approved by University of Missouri at Kansas City-Institutional Animal Care and Use Committee (UMKC-IACUC) (Permit # A3397-01) in accordance with the National Institutes of Health (NIH) guidelines. All experiments using mice in this section were conducted under isoflurane anesthesia, and all efforts were made to minimize suffering. A novel model of oxidant-mediated “sterile” orofacial inflammatory pain was produced by intra-masseter muscle (IMM) injection of PPC, which decays readily to the same reactants that are produced by activated phagocytes [28]. We used this model to examine the *in vivo* effects of EUK-134, a synthetic serum-stable superoxide dismutase/catalase mimetic (SOD/CATm) agent that can attenuate prooxidant injury [29].

Under isoflurane anesthesia, four (4) groups of mice [Group I: EUK-134 vehicle/Normal saline (NS) control; Group II: EUK-134 vehicle/PPC; Group III: EUK-134 (10 nmol/mouse)/NS; Group IV: EUK-134 (10 nmol/mouse)/PPC)] received a single IMM injection of PPC or normal saline (NS) (as control). The main objective here was to determine whether PPC would in any way modulate the expression of TLR4 in an orofacial model of inflammatory pain. Injections were made at a dose of 250 pmol of PPC in NS/mouse or NS alone as control, in a volume of 35 µl via a Hamilton syringe with a 30-gauge needle. All injections were made in the left caudal region of the right masseter muscle to ensure that PPC is confined to the muscle tissue. Where applicable, EUK-134 (10 nmol/mouse) in NS or NS as control, was also administered IMM in the same masseter 1 h before PPC. Thereafter, the same dose of EUK-134 was given once daily to applicable groups under the same conditions at the same time of day.

### Animal Behavioral Assessment

In this procedure, the experimenter was blinded to all treatments. Changes in pain behavioral responses were measured under 0.25% isoflurane anesthesia, which was established when animals showed withdrawal responses to a tail pinch with a clipper calibrated to produce 400 g of force. Prior to any treatment, a baseline mechanical threshold (at t = d0) for eliciting the head withdrawal responses was determined in all test groups over 3-training sessions on 2 successive days. Mice were tested for their responses to mechanical stimulation in the masseter muscle region according to modified methods of Imbe et al [30] and Miyamoto et al [31]. The test results were recorded with a calibrated Electronic von Frey™Anesthesiometer (IITC Inc., Woodland Hills, CA).

Mechanical head withdrawal threshold [MHWT] was assessed at 0-, 0.25-, 1-, 2- and 7-d after NS, EUK-134 and/or PPC. The probe was applied to the skin above the masseter but avoiding the whisker pad area, beginning with the ipsilateral side first, followed immediately by the contralateral side. Mechano-allodynia was recorded as an active withdrawal of the head or vigorous snout flinching to this normally non-noxious stimulus. Decreases in mechanical threshold to display head withdrawal were considered indicative of mechanical hyperalgesia. The baseline value was determined before drug treatment in all test groups by averaging the mechanical head withdrawal threshold (MHWT) for ten consecutive mechanical stimulations applied at 1-min intervals. The force in Newton (1N = 102 gram-force) needed to elicit a withdrawal of the head was recorded following five stimulus presentations of 1-s duration at 1-min intervals. The average of these five values was used as the withdrawal threshold. Post-injection MHWT was normalized to the baseline value in order to make comparisons across subjects and experimental groups. Percent changes from the baseline were calculated and plotted against time.

As an objective measurement, MHWT permitted a functional assessment of pain sensitivity and its treatment outcomes. Immediately before IMM EUK-134 (10 nmol), NS and/or PPC, MHWT was measured for each mouse.

### Biochemical Measurements in Animals

TLR4 expression and cytokine levels were determined in masseter tissue punches taken with 3-mm diameter tissue punch with a plunger (Braintree Scientific, Braintree, MA). At the end of each treatment, animals were killed by cervical location and exsanguination, and the carcasses immediately packed in ice. Tissue punches were obtained from ice-cooled carcasses not more than 5 min after animals were killed. Punches were taken from relatively the same five (5) different locations from ipsi- and contra-lateral masseter per mouse. The tissue samples obtained from the ipsi-lateral masseter were combined, as were those from the contralateral side. Samples were immediately homogenized and extracted in tissue protein extraction lysis buffer (Tissue-PE LB™) (G Biosciences, St. Louis, MO) supplemented with EDTA, dithiothreitol (DTT), protease inhibitors and 0.1% Igepal. Tissue homogenates were centrifuged at 16,500×-g at 4°C for 20 min to remove tissue/cellular debris. The supernatants were divided into two portions, and stored at −80°C for future use in immuno-blots and ELISA.

We conducted immuno-blots with anti-TLR4 IgG polyclonal antibody (pAb) (Abcam, Cambridge, MA) to determine TLR4 expression levels.

We used ELISA kits (Thermo Scientific/Pierce, Rockford, IL) to quantify the levels of TNF-α and IL-1β according to the manufacturer’s instruction manuals. Bicinchoninic acid (BCA) protein assay kit (Thermo Fisher/Pierce, Rockford, IL) was used to determine total protein contents with bovine serum albumin (BSA) as a standard.

### Statistical Analysis

Statistical Package for the Social Sciences (SPSS) and GraphPad Prism Software (San Diego, CA) were used to perform data analysis for *in vitro* and *in vivo* experiments, respectively. All data are presented as mean ± SEM from at least 3–9 independent experiments conducted in duplicates, where applicable, and analyzed by 1- or 2-way analysis of variance (ANOVA) followed by Tukey’s (*in vitro*) or Fisher’s PLSD (*in vivo*) post hoc tests. Significance was assigned at p≤0.05%.

## Results

### Effect of Pro-oxidants on the Viability of HEK-Blue mTLR4 Cells

The viability of HEK-Blue mTLR4 cells was initially determined for each pro-oxidant over a wide range of five concentrations at a 24 h period. We eventually used sub-threshold prooxidant concentrations in all subsequent experiments in for each oxidant in which cell survival was ≥93%. This careful approach was necessary to obviate potential adverse effects that could result from oxidant toxicity. Thus, at 1 µM and 5 µM of PPC, 97.0 and 94.0% of cells were viable, respectively, but at 10 µM only 76% cells were viable. At 50 µM PDO, 93% of cells were viable, but at 1 mM PN, 98% of cells were viable. For each oxidant, at least 5 independent experiments were performed in duplicate.

### MPLA, a TLR4-specific agonist, Induced Robust SEAP Release in HEK-Blue mTLR4 Cells

**Figure 1:**
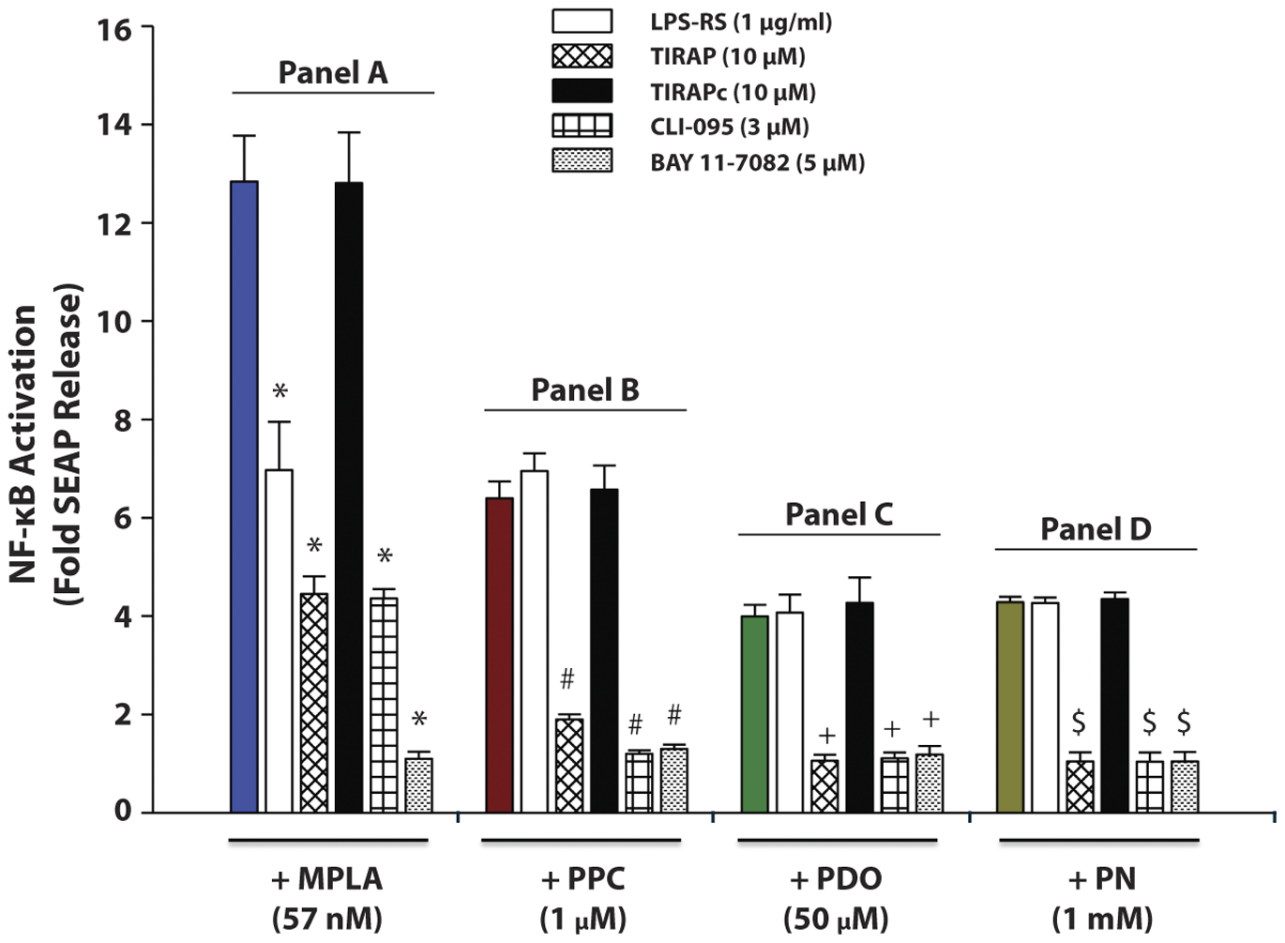
Effect of various inhibitors on secreted embryonic alkaline phosphatase (SEAP) release in monophosphoryl lipid A (MPLA) - and oxidant-induced HEK-Blue mTLR4 activation. Cells were pre-incubated with various inhibitors for 30 min followed by incubation with MPLA (57 nM), PPC (1 µM), PDO (50 µM) or PN (1 mM) for 16 h in continued presence of the inhibitors. SEAP release in the supernatant was determined using Quanti-Blue, and the absorbance read at 650 nm. The color-coded column in each panel (A to D) represents treatment with MPLA, PPC, PDO or PN alone, respectively, in the absence of TLR4 antagonists/inhibitors. The data represent 5 independent experiments; *p≤0.01 versus treatments with MPLA alone, or [TIRAPc+MPLA]; ^#^ *p*≤0.001 vs treatments with PPC alone, [LPS-RS +PPC], and [TIRAPc +PPC]; ^+^ *p*≤0.001 vs treatments with PDO alone, [LPS-RS +PDO], or [TIRAPc+PDO]; ^$^ *p*≤0.001 vs treatments with PN alone, [LPS-RS +PN], or [TIRAPc +PN].

Treatment with 100 ng/ml (57 nmol) of MPLA for 16 h induced SEAP release by a mean of 12.8 fold over the same cells under the same treatment conditions, but in the absence of MPLA. Cell pre-treatment with LPS-RS (a potent TLR4 antagonist), TIRAP (an adaptor protein inhibitor in the TLR4 signaling pathway), CLI-095 (a TLR4 signaling inhibitor), or BAY11-7082 (an irreversible inhibitor of IκB-α phosphorylation that results in the inactivation of NF-κB) for 30 min prior to stimulation with MPLA decreased SEAP release by 53, 73, 74 and 98%, respectively. Pretreatment with TIRAP control (TIRAPc), a scrambled nonfunctional sequence of TIRAP, prior to MPLA had no effect on SEAP release (Fig. 1, Panel A).

### Pro-oxidants Increased TLR4 Signaling in HEK-Blue mTLR4 Cells

Treatment with PPC (1 µM) for 16 h induced SEAP release by 6.4 fold over untreated control cells incubated under the same conditions. Pre-treatment with TIRAP, CLI-095 or BAY11-7082 for 30 min prior to treatment with PPC decreased SEAP release by 86.3, 97.4, and 95.3%, respectively. However, LPS-RS and TIRAPc had no effect on PPC-induced SEAP release (Fig. 1, Panel B).

Treatment with 50 µM of PDO for 16 h induced SEAP release by 3.99 fold over the same cells in the absence of PDO. Pre-treatment with TIRAP, CLI-095 or BAY11-7082 for 30 min prior to PDO treatment decreased SEAP release by 98.7, 97.4, and 98.2%, respectively. LPS-RS and TIRAPc had no effect on PDO-induced SEAP release (Fig. 1, Panel C).

Treatment with 1 mM PN for 16 h induced SEAP release by 4.28 fold over untreated control cells without PN, but under the same treatment conditions. Pre-treatment with TIRAP, CLI-095, or BAY11-7082 for 30 min prior to PN treatment decreased SEAP release by 99.5, 99.3, and 99.5%, respectively. Again, LPS-RS and TIRAPc did not affect PN-induced SEAP release (Fig. 1, Panel D).

Overall, our data indicate that pro-oxidants act through the TLR4 signaling, but curiously in all cases pretreatment with either LPS-RS or TIRAPc did not affect PPC-, PDO- or PN-induced SEAP release. All the prooxidants used in this study induced SEAP release at quantitatively lower levels (Fig. 1) compared to MPLA, a TLR4-specific agonist. However, PPC produced the most robust release of SEAP compared to the other pro-oxidants, which suggests the most enhanced NF-κB activation by PPC.

### Anti-mTLR4 Antibody Neutralized the Activation of TLR4 and PPC

**Figure 2:**
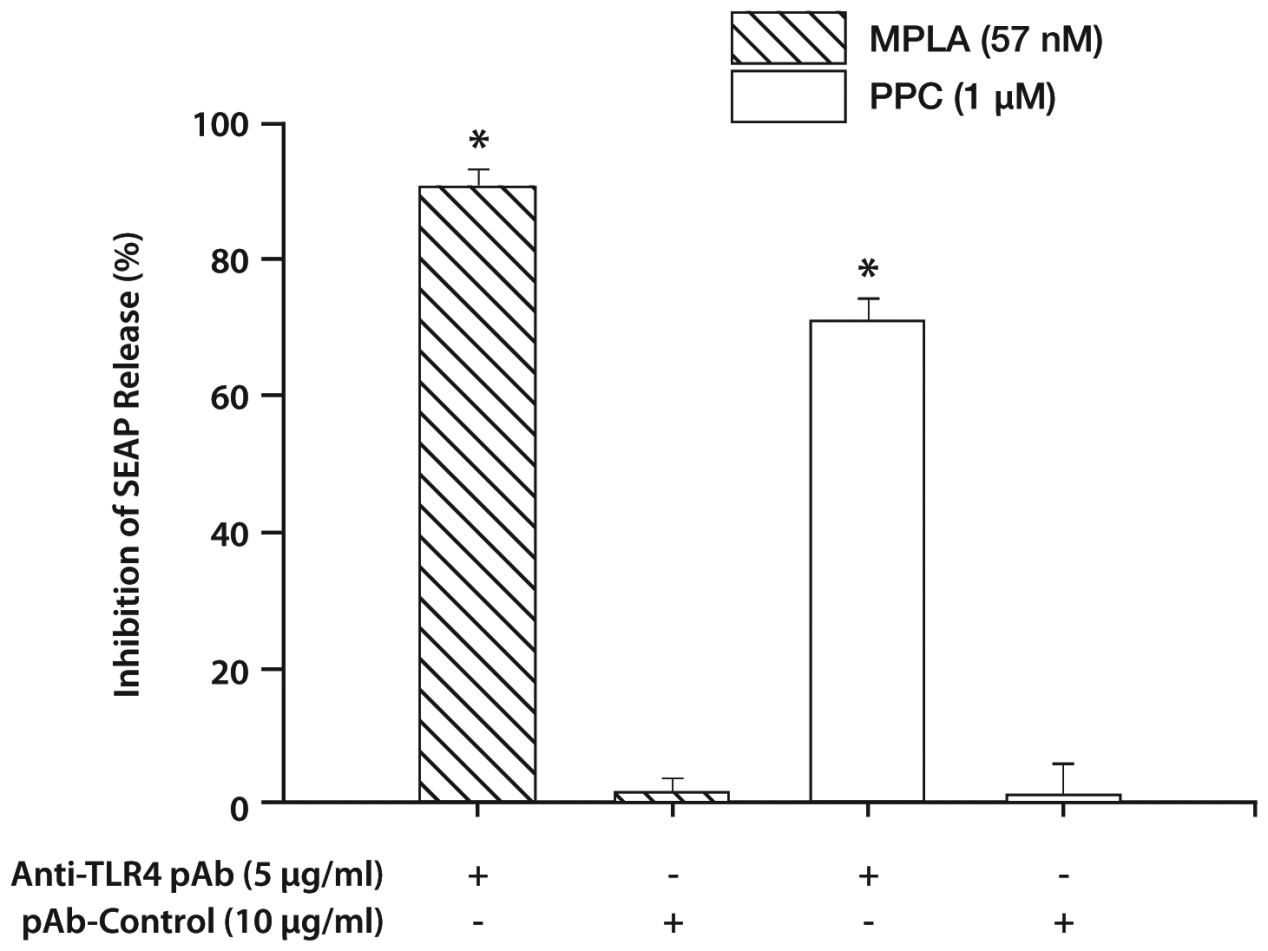
Effect of anti-TLR4 pAb on secreted embryonic alkaline phosphatase (SEAP) release in MPLA and pro-oxidant-stimulated HEK-Blue mTLR4 cells. Cells were preincubated with anti-TLR4 pAb (5 µg/ml) for 2 h followed by stimulation overnight with either MPLA (57 nM) or PPC (1 µM). Preincubation with rat polyclonal IgG (10 µg/ml) was used as control. SEAP release in the supernatant was determined using Quanti-Blue with the absorbance read at 650 nm. The data represent 3 independent experiments. *p≤0.001 vs treatments with MPLA or PPC preincubated with anti-TLR4.

Preincubation of HEK-mTLR4 cells with an anti-TLR4 pAb followed by stimulation with either MPLA or PPC, inhibited SEAP release induced by either of the agents by 90% and 71%, respectively (Fig. 2). Preincubation with rat polyclonal IgG control had no effect on SEAP release mediated by either MPLA or PPC. The results confirmed that SEAP release was as a result of TLR4 activation, and that both MPLA and PPC activated the receptor system.

### Antioxidants (Ebselen and PBN) Decreased MPLA- and Pro-oxidant-induced SEAP Release in HEK-Blue mTLR4 Cell

**Figure 3:**
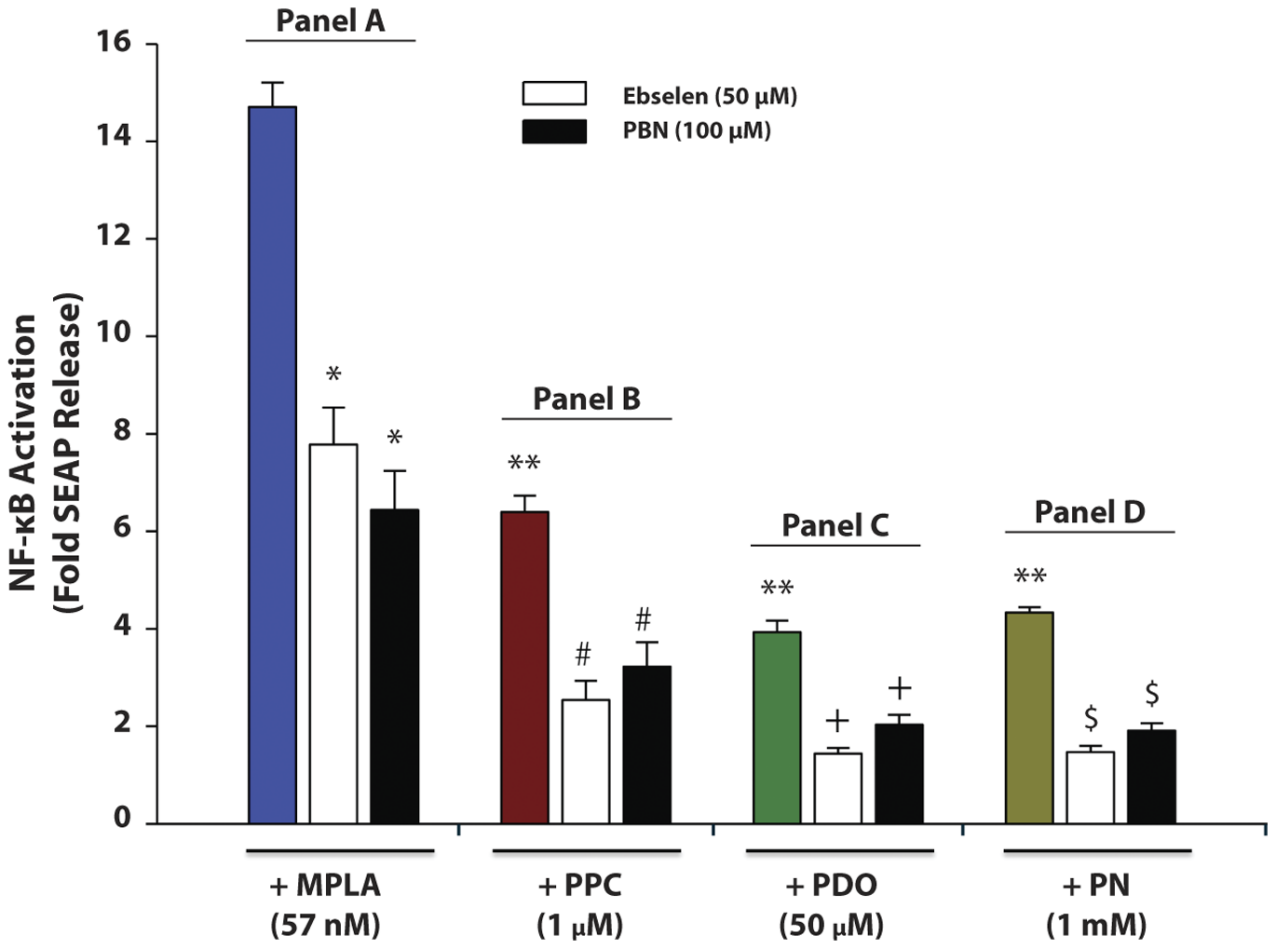
Effect of anti-oxidants on secreted embryonic alkaline phosphatase (SEAP) release in pro-oxidant-stimulated HEK-Blue mTLR4 cells. Cells were pre-treated with Ebselen (50 µM) or PBN (100 µM) followed by stimulation with MPLA (57 nM), PPC (1 µM), PDO (50 µM) or PN (1 mM) for 16 h in the continued presence of the anti-oxidants. SEAP release in the supernatant was determined using Quanti-Blue, and absorbance was read at 650 nm. The color-coded column in each panel (A to D) represents treatment with MPLA, PPC, PDO or PN alone, respectively, in the absence of the anti-oxidants. [The data represent n = mean ± SEM in 6 independent experiments; *p≤0.01 compared to MPLA treatment alone; **p≤0.001 compared with MPLA treatment alone; ^#^ *p*≤0.001 vs treatment with PPC alone; ^+^ *p*≤0.001 vs treatment with PDO alone; ^$^ *p*≤0.001 vs treatments with PN alone].

Pre-treatment with free radical scavengers Ebselen and PBN decreased MPLA-induced SEAP release by 66 and 50%, respectively (Fig. 3, Panel A). Pretreatment with Ebselen and PBN also decreased PPC-mediated SEAP release by 75 and 65.6%, respectively (Fig. 3, Panel B), and also reduced PDO-induced SEAP release by 87.9 and 77.9%, respectively (Fig. 3, Panel C). In addition, pretreatment with the antioxidants decreased PN-mediated SEAP release by 93 and 82%, respectively (Fig. 3, Panel D). Our data confirm that prooxidants can stimulate TLR4 as in all cases shown here. In addition, the antioxidants significantly decreased SEAP release induced by both MPLA and prooxidants. The data confirm that ROS/RNS are important in activating TLR4 signaling.

### Effect of MPLA and Pro-oxidants on HEK-Blue mTLR2 Cell Activation

**Figure 4:**
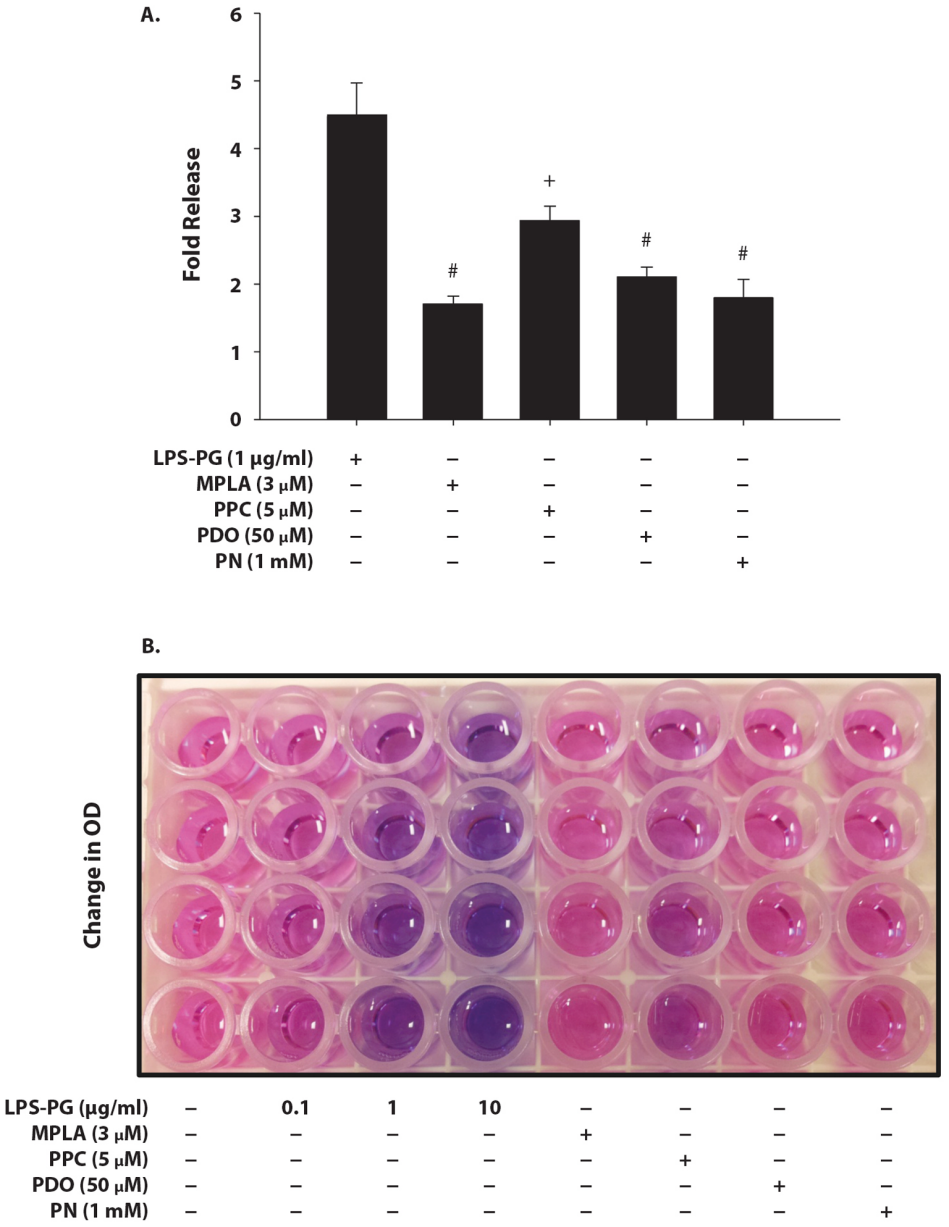
Effect of MPLA and pro-oxidants on levels of SEAP release from HEK-Blue mTLR2 cells. Cells were stimulated with 1 µg/ml of LPS from *Porphyromonas gingivalis* (LPS-PG) (1 µg/ml), MPLA (57 nM), PPC (1 µM), PDO (50 µM), or PN (1 mM) for 16 h. SEAP released into the supernatant was quantified using Quanti-Blue (Fig. 4A) [n = means ± SEM from 5 independent experiments; ^#^ *p*≤0.01 vs treatments with LPG-PG or PPC alone; ^+^ *p*≤0.01 versus treatment with LPS-PG alone]. **Fig. 4B** - A representative qualitative representation of LPS-PG concentration-dependent changes in purple/blue color development (absorbance) indicative of increased SEAP release (i.e., enhanced with increasing NF-κB) with increasing LPS-PG concentration after Quanti-Blue reaction. HEK-Blue mTLR2 cells incubated with different concentrations of LPS-PG at 0.1, 1.0 and 10 µg/ml for 16 h were compared to treatments with different prooxidants assumed to be potential TLR2 activators.

Treatment with 1.0 µg/ml of LPS-PG (a specific agonist for TLR2) for 16 h induced SEAP release by 4.5 fold. MPLA, PDO and PN at the concentrations that produced robust SEAP release following TLR4 stimulation did not induce a significant SEAP release in TLR2 with only 1.7-, 2.1- and 1.8-fold release, respectively. However, PPC at a higher concentration (5 µM) increased SEAP release by 2.9 fold (Fig. 4A), showing that TLR2 can be stimulated at a high prooxidant concentration. In addition, LPS-PG increased SEAP release in a concentration-dependent manner (Fig. 4B).

### Effect of PN on Protein Tyrosine Nitration in HEK-Blue mTLR4 and HEK-Blue mTLR2 Cells

**Figure 5:**
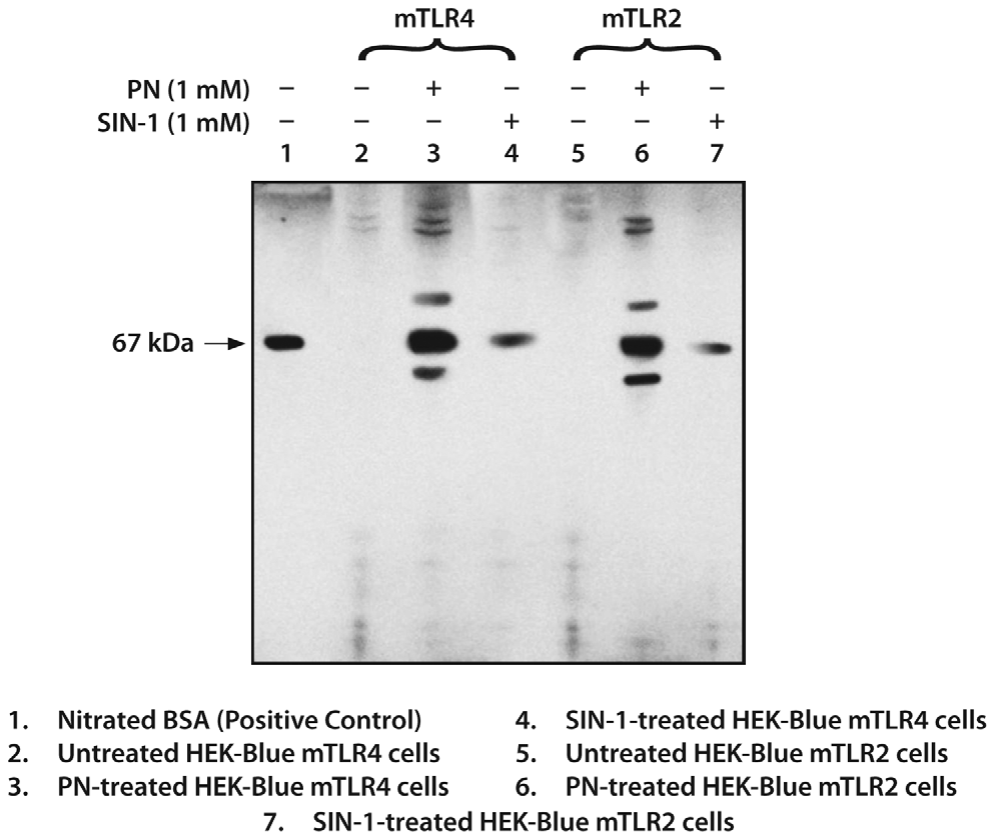
Representative immunoblots of nitrated proteins following incubation of HEK-Blue mTLR4 and HEK-Blue mTLR2 cells with an equimolar concentration (1 mM) of either PN or SIN-1 for 3 h. Cell lysates were subjected to immunoblot using anti-nitrotyrosine. BSA nitrated at the tyrosine residue(s) was used as positive marker for protein nitration.

To determine the effectiveness and kinetics of PN in nitrating tyrosine protein residues, we treated cells with PN or SIN-1 at equimolar concentration of 1 mM under the same conditions. Treatment with PN produced multiple bands confirming nitration of different proteins in both cells. We used SIN-1, an effective nitric oxide (NO) donor [32], as a comparative positive control for protein tyrosine nitration (Fig. 5). Treatment with SIN-1 produced a single band of nitrated protein indicating that both PN and SIN-1 were effective in generating nitrated proteins, but at different kinetic rates. From the electrophoresis profiles, PN appears to have nitrated the same set of tyrosine residues in both cells. Nitrated bovine serum albumin was used as positive marker of nitrated proteins.

### Effect on HEK-Blue Null1-v Cells

**Figure 6:**
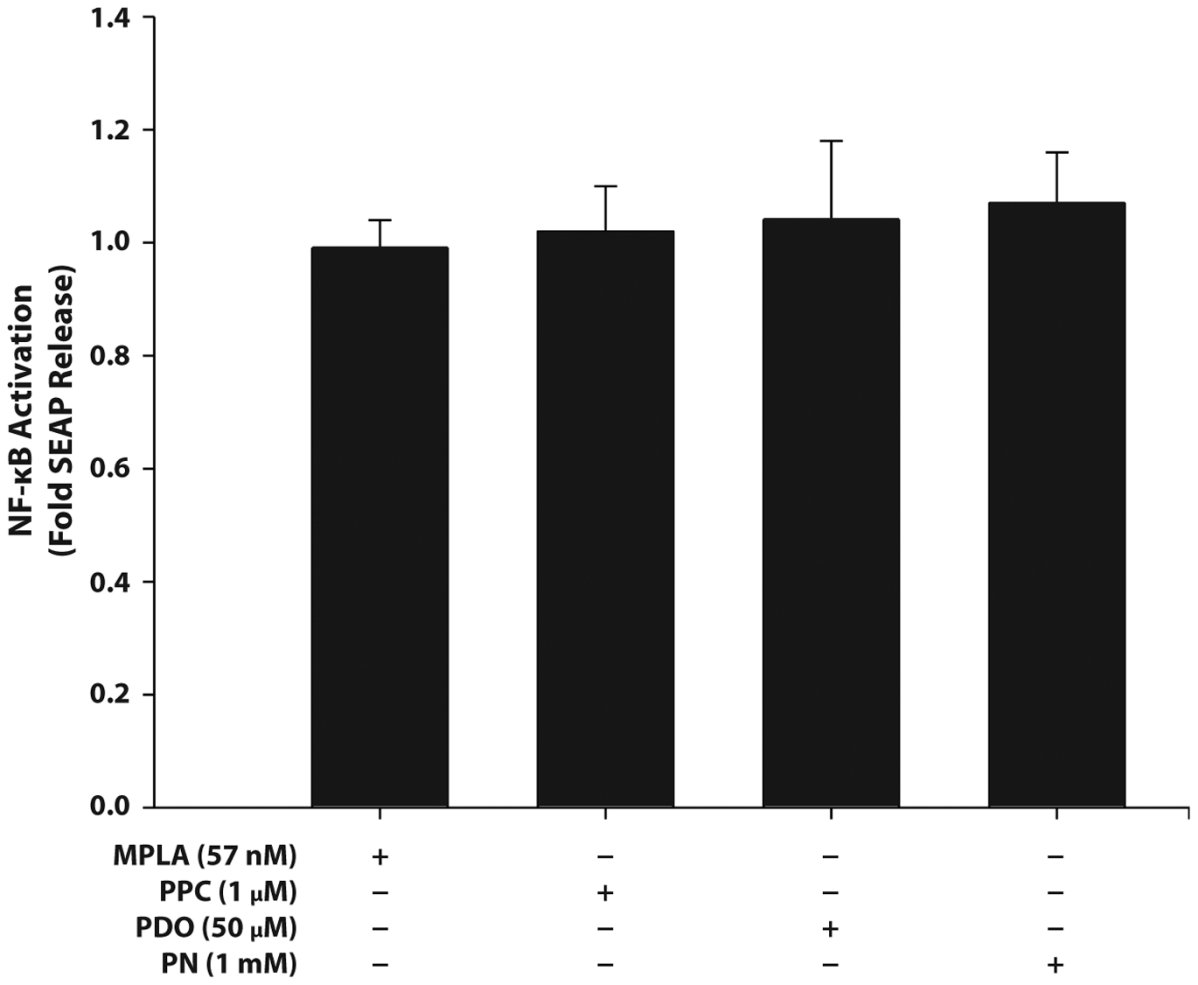
Effect of pro-oxidants on secreted embryonic alkaline phosphatase (SEAP) release in HEK-Blue null 1-v cells. Cells were stimulated with MPLA (57 nM), PPC (1 µM), PDO (50 µM), or PN (1 mM) for 16 h. The SEAP levels in the cell supernatant were determined using Quanti-Blue with the absorbance was read at 650 nm. The data are presented as means ± SEM from at least 5 independent experiments.

Neither MPLA nor any of the pro-oxidants significantly induced SEAP release from HEK-Blue null-v cells (Fig. 6), which confirmed the inactivity of the reporter gene insert.

### PPC Increased Inflammatory Response in Mice

A single IMM injection of PPC produced low-grade swelling of the injected side (ipsilateral) in ∼ 5 h indicative of an inflammatory response. This visual sign of inflammation persisted for over 21 days. Examination of hematoxylin- and eosin-stained cryo-sections (15 µm) of the masseter muscle at d 7 after PPC (data not shown) revealed cystic lesions and infiltration of lymphoid cells, which provided histological evidence of masseter inflammation. Injected and un-injected animals showed no differences in grooming and exploration. Injected mice did not have any difficulty in feeding, and gained weight comparable to the un-injected animals.

### PPC Increased the Head-withdrawal Threshold to Mechanical Stimulation Quantified as Mechanical Head Withdrawal Threshold (MHWT)

**Figure 7:**
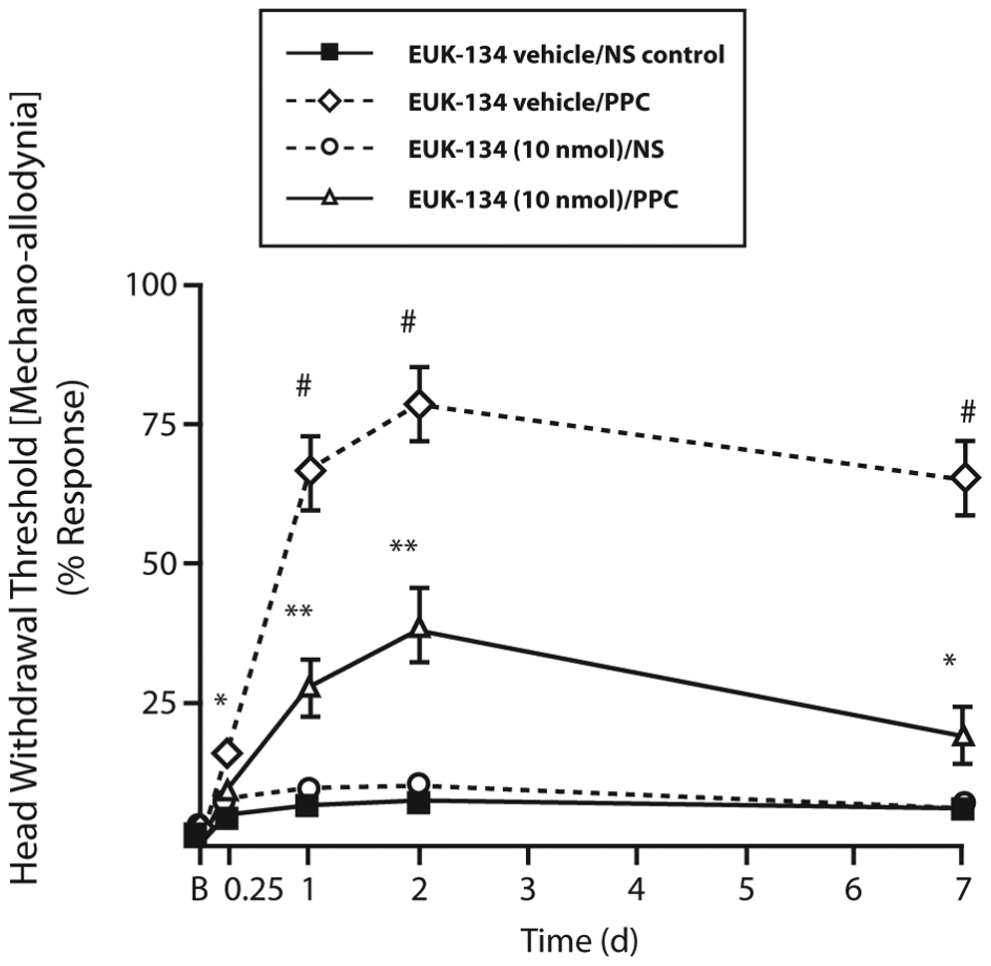
Percentage (%) responses in mechanical head withdrawal threshold (MHWT) to a von Frey filament following PPC-induced inflammation in the mouse masseter muscle at d0, d0.25, d1, d2 and d7. [n = 5–9 mice/group; ^#^ *p*≤0.01 compared to all test groups; **p = 0.001 compared to [EUK-134 vehicle/NS] and [EUK-134 (10 nmol)/NS] groups; *p = ≤0.01 compare to [EUK-134 (10 nmol)/NS] on d1 and d7 [2-way ANOVA (group and time)/Fisher’s PLSD post hoc test].

Mechanical allodynia was present ∼5 h after PPC in phase with the appearance of inflammatory response of swelling, with no changes observed before PPC (t = d 0) in all test groups. Pretreatment with [EUK-134 (10 nmol) in the ipsilateral masseter and NS (in the contralateral side) 1 h before PPC significantly reduced PPC-induced mechanical hypersensitivity quantified as MHWT by 39, 40 and 41% on d 1, d 2 and d 7, respectively (Fig. 7). EUK-134 alone had no effect on MHWT in the time-course suggesting that the test agent was not anti-nociceptive in the absence of underlying inflammation. No secondary mechanical allodynia was observed in the contralateral masseter, which suggests that the inflammatory response was limited to the injected masseter over the testing period.

### PPC Increased the Expression of TLR4 in the Masseter Muscle

**Figure 8:**
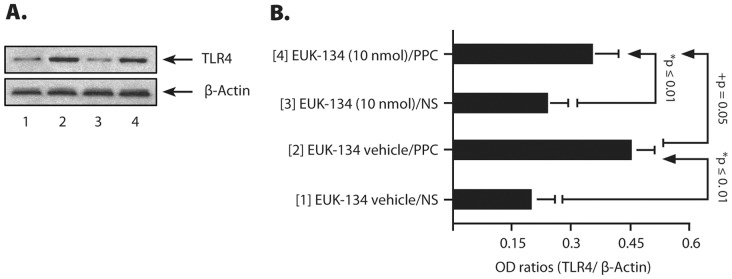
A representative masseter TLR4 expression levels in different treatment groups (A) at d7 after treatments with EUK-134 (10 nmol/mouse), NS and/or PPC (250 pmol/mouse). The histograms (**B**) represent the optical density (OD) ratios of TLR4 immunoblot signals normalized to those of β-actin from the same test groups. The numbers in parenthesis in (**B**) are related to the immuno-blot signals in (**A**) [n = 5–6 mice/group; *p ≤ 0.01 compared with [EUK-134 (10 nmol)/NS]- and [EUK-134 vehicle/NS]-treated group; **^+^** *p* = 0.05 versus [EUK-134 vehicle/PPC] [1-way ANOVA/Fisher’s PLSD post hoc test].

IMM treatment with [EUK-134 vehicle/PPC] up-regulated TLR4 expression in the masseter muscle by 2.5-fold compared to [EUK-134 vehicle/NS] and [EUK-134 (10 nmol)/NS] (Fig. 8A). Treatment with [EUK-134 (10 nmol)/PPC] decreased TLR4 expression by 35% compared with [EUK-134 vehicle/PPC] treatment (Fig. 8B). This suggests that increase in TLR4 protein expression could only be due to either PPC and/or its decay products, which was attenuated in the presence of EUK-134 (10 nmol). Notably, treatment with [EUK-134 (10 nmol)] alone had no effect on TLR4 expression. PPC increased TLR4 expression, which indicates, for the first time, that exogenous oxidants can up-regulate TLR4 expression and can potentially dysregulate innate immune responses.

### PPC Increased Tissue Levels of TNF-α and IL-1β in the Masseter Muscle

**Figure 9:**
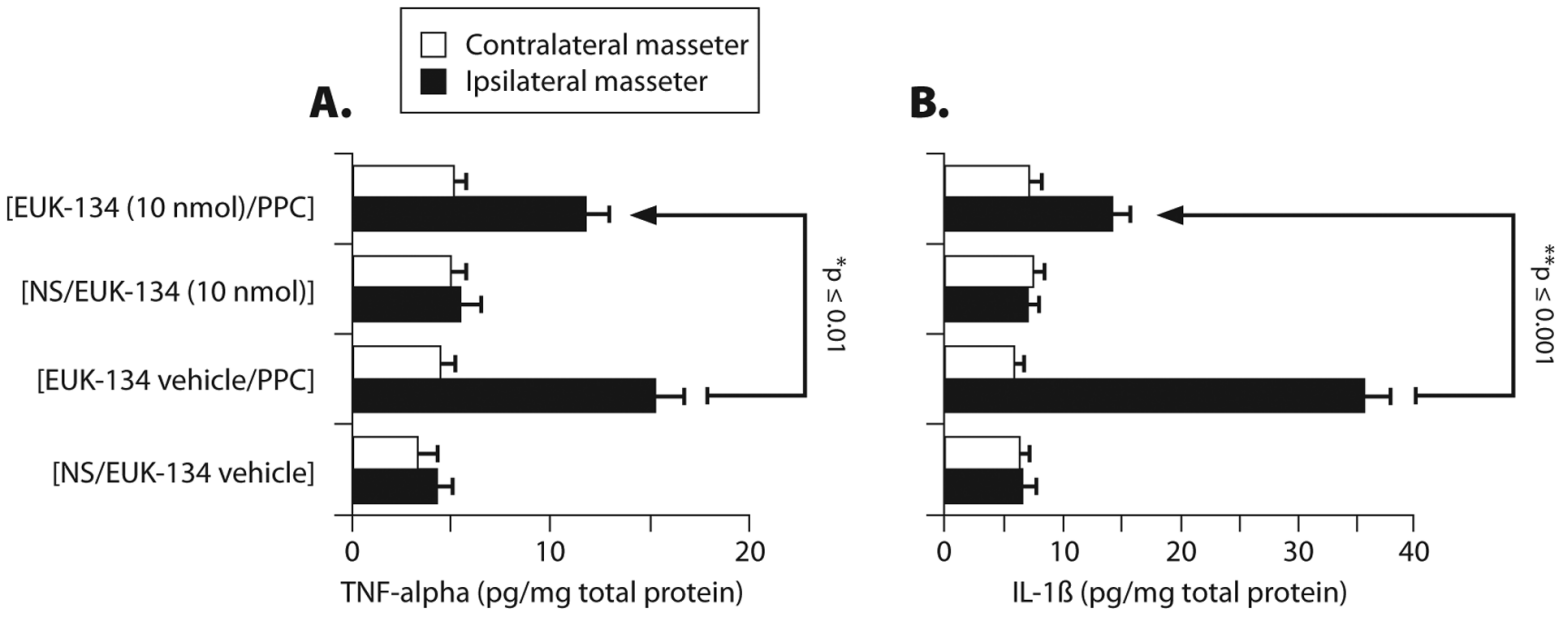
Masseter muscle tissue levels of (A) TNFα and (B) IL-1β at d7 following different treatments. Values were normalized to total tissue protein levels. [n = 5–6 mice/group; *p≤0.01, **p≤0.001 compared with [EUK-134(10 nmol/mouse)/PPC]-treated group for each cytokine.

PPC increased intra-masseter levels of TNFα and IL-1β by 3.6- and 5.4-fold, respectively, on d7 after PPC compared to [NS/EUK-134 (10 nmol/mouse)]- or [NS/EUK-134 vehicle]-treated group, respectively (Fig. 9A & 9B). EUK-134 (10 nmol/mouse) reduced PPC-mediated increases in TNF-α and IL-1β by 24% and 60%, respectively, and was more effective in reducing tissue levels of IL-1β than those of TNF-α at the same time interval after PPC. In this oxidant model, TNF-α tissue profile appears to be more persistent than that of IL-1β in maintaining PPC-induced inflammation.

Furthermore, PPC-mediated increases in TNF-α and IL-1β were only present in the inflamed ipsilateral masseter with no changes in basal levels on the contralateral side. The data correlate with changes in pain sensitivity quantified as MHWT (Fig. 7A), and suggest a functional linkage between MHWT and increases in intra-masseter TNF-α and IL-1β levels.

## Discussion

Our present *in vitro* results have shown that exogenous pro-oxidants can activate NF-κB through stimulation of TLR4 or TLR2 to induce SEAP release. We have used three sources of prooxidants at sub-threshold concentrations namely potassium peroxychromate (PPC), potassium dioxide (PDO) and sodium peroxynitrite (PN). We chose each agent based on the primary prooxidant that each would theoretically produce in aqueous medium. We used these agents to determine to what extent the prooxidants they produce may be ‘sensed’ through TLR4 or TLR2 expressed on cell surface to activate NF-κB in HEK-Blue cells stably transfected with mTLR4 or mTLR2.

Comparing the effects of these prooxidants on SEAP release with their effects on MPLA- and LPS-PG-induced SEAP release, we have demonstrated that prooxidant activation of TLR4 was by far more robust than that of TLR2. MPLA and LPS-PG are specific agonists for TLR4 and TLR2, respectively. All the prooxidants used in this study increased SEAP release at quantitatively different levels (Fig. 1, Panels B, C & D) following TLR4 stimulation, which, in effect, is evidence of enhanced NF-κB activation. The implication of this effect is the potential to increase the synthesis and release of proinflammatory cytokines and chemokines in the tissue, which is supported by our *in vivo* data.

Among the prooxidants used, PPC produced the most robust effect at a much lower concentration than PDO and PN. This could result from the fact that both *in vitro* and *in vivo*, PPC is capable of producing robust reactive species of oxygen for an extended period that can cause cellular lipid peroxidation [33], a hallmark of oxidative damage. We have also shown that IMM PPC as a model of orofacial pain in mice evoked mechanical allodynia and increased TLR4 expression in the masseter tissue, which were attenuated by EUK-134, a synthetic serum-stable scavenger for oxidative species [29]. Decomposing PPC has previously been used as a source of ROS to examine its effects on cellular functions [34], [35]. Among the various oxidation states of chromium (Cr) from (−II) to (+VI), the peroxychromate anion [Cr0_8_]^3−^ consisting of a central chromium atom in the oxidation state of (+V) can decompose spontaneously in neutral or alkaline aqueous solutions. The peroxychromate anion decomposes readily in aqueous systems to release several reactive species including hydrogen peroxide, hydroxyl radical, singlet oxygen, and possibly superoxide [33]. Furthermore, PPC has been used as an oxidant-induced injury model to study arthritis [36]. The products of aqueous decay of PPC have been shown to activate the oxidative burst of phagocytes in human blood [37]. The choice of a Cr salt with (+VI) valency state was therefore based on its ability to generate a robust amount of prooxidants in biological systems, and not based on its clinical utility as a trace metal element per se. None of the other clinically relevant transition trace metals such as iron (Fe) or copper (Cu) in any of their respective valency states could replace chromium (+VI) because of its fidelity in generating reactive molecules *in vivo* and *in vitro*.

A solution of PDO in DMSO and 18-crown-6-ether was used as a source of generating a protected pure superoxide anion *in situ*. In aqueous solution, spontaneous disproportion of superoxide leads rapidly to the formation of H_2_O_2_. Both oxygen species are precursors to the most powerful oxidant hydroxyl radical. Similarly, Lorne, et al [38] showed that generation of superoxide through direct interaction of xanthine oxidase with TLR4 resulted in enhanced activation of NF-κB and production of NF-κB dependent proinflammatory cytokines.

Peroxynitrite (PN) anion (ONOO-) is a short-lived oxidant species that is produced by the reaction of nitric oxide and superoxide [39], [40]. PN can cause a multitude of biological effects with resultant adverse actions on cell viability and function [41], [42]. Injection of PN into the mouse hind paw has been shown to induce significant hyperalgesic responses [43]. In addition, neutralization of superoxide (as a precursor of PN), or treatment with the PN scavenger, uric acid, suppressed inflammatory pain responses in a number of rodent models [44], [45]. Nitrated tyrosine residues represent biological markers for PN-induced tissue damage and have been detected in inflamed [46] and in aged tissues [47], [48]. In the present study, we examined the effectiveness of PN in HEK-Blue mTLR4 and HEK-Blue mTLR2 cells by performing immunoblot of potential proteins nitrated at the tyrosine residues after a short-term incubation with PN (Fig. 4). The presence of multiple unidentified protein bands in the immuno-blot confirmed the efficacy of PN-mediated protein nitration at the tyrosine residues. Moreover, PN (1 mM) was more effective in producing nitrated proteins than equimolar concentration SIN-1, an agent that generates and donates PN *in situ* [49].

To explore the signaling mechanism(s) for inducing SEAP release, we used various inhibitors that act at different levels of signal transduction pathway after TLR4 activation. LPS-RS specifically inhibited only MPLA- but not PPC-, PDO- or PN-induced SEAP release in HEK-Blue mTLR4 cells. This result confirmed a specific affinity of LPS-RS for MPLA-mediated effects through activation of TLR4. LPS-RS, obtained from the photosynthetic bacterium *Rhodobacter sphaeroides*, is a potent antagonist of toxic LPS in both human and murine cells, which can also prevent LPS-induced shock in mice [50]. Nonetheless, a neutralizing antibody directed against TLR4 inhibited responses to both MPLA and PPC. This confirmed that both MPLA, a specific agonist for TLR4, and prooxidants utilized TLR4 to induce SEAP release i.e., they use the same receptor system.

LPS-RS employs two distinct mechanisms to block LPS-dependent activation of TLR4. The main mechanism appears to consist of a direct competition between the pentaacylated LPS (LPS-RS) and the hexaacylated LPS or MPLA for the same binding site on the MD2 co-receptor. The secondary mechanism involves the ability of LPS-RS/MD2 complexes to inhibit the function of MPLA/MD2 complexes on the TLR4 [51], [52]. The ineffectiveness of LPS-RS to inhibit PPC-, PDO- or PN-induced SEAP release (Fig. 1, Panels 1B, 1C and 1D) compared with its inhibition of MPLA-induced SEAP release could be due to the inability of LPS-RS to complex with prooxidants. In view of the robust level of SEAP inhibition following PPC in the neutralization data (Fig. 2), our results would suggest that pro-oxidants may act through both the extra- and intra-cellular domains of TLR4 to modify the receptor function. Another possible mechanism is that prooxidants may enhance the dimerization of TLR4 to MD-2 [53], or induce a structural change in the dimerized TLR4 receptor complex to decrease the recruitment of the adaptor proteins to the intracellular domain of TLR4 [54]. These may help explain the distinct effects observed after the receptor was exposed to MPLA, LPS-RS and prooxidants.

Therefore, to further examine the action of pro-oxidants through the intracellular domain of TLR4, we used TIRAP inhibitor and CLI-095. TIRAP, an adapter molecule in the TLR signaling pathway, has been shown to function downstream of TLR4 [55]. The importance of TIRAP in TLR4 signaling was shown in a report that TIRAP-deficient mice had defects in cytokine production and in activation of the NF-κB and mitogen-activated protein kinases (MAPK) in response to LPS, a native ligand for TLR4 [56]. Furthermore, LPS stimulation of TLR4 did not induce NF-κB activation in RAW cells pre-treated with the TIRAP peptide and, no effect on NF-κB activation was observed upon pretreatment with a control peptide in which the TIRAP sequence was reversed [57]. Thus, pretreatment with TIRAP peptide inhibitor blocked the effect of LPS-mediated activation of TLR4, whereas TIRAPc did not have any inhibitory effect on TLR4 activation.

Consistent with this conclusion, our results demonstrated that only TIRAP peptide but not the control TIRAP with reversed sequence (TIRAPc) inhibited the MPLA-, PPC-, PDO- or PN-induced SEAP release through TLR4 stimulation. CLI-095, also known as TAK-242, is a novel cyclohexene derivative that suppresses TLR4 signaling by inhibiting the production of NO and pro-inflammatory cytokines [58]. CLI-095 acts by blocking signaling mediated by the intracellular domain of TLR4, especially its adaptor protein molecules [59]. Cell pre-treatment with CLI-095 inhibited MPLA-, PPC-, PDO- or PN-induced SEAP release through TLR4. Next, we used BAY11-7082 to examine the effect of pro-oxidants at the NF-κB activation level, which is the point of convergence for both MyD88-dependent and MyD88-independent pathways in TLR4 activation. BAY11-7082 is an irreversible inhibitor of IκB-α phosphorylation resulting in the inactivation of NF-κB [60]. Pretreatment of cells with BAY11-7082 not only inhibited MPLA-, but also all the pro-oxidant-induced SEAP release through TLR4 stimulation. This feature shows that BAY11-7082 has a potential as a potent antioxidant/anti-inflammatory agent.

To further clarify the action of pro-oxidants, we examined the effect of two free radical scavengers, Ebselen and PBN, against SEAP induction by MPLA and pro-oxidants through TLR4 activation. Ebselen, a novel organo-selenium compound and glutathione peroxidase mimetic [61], [62], is a potent scavenger of hydrogen peroxide as well as peroxynitrite and hydroperoxides including membrane bound phospholipid and cholesterylester hydroperoxides [63]. Therefore, Ebselen may represent a new class of anti-inflammatory agent in the treatment of hydroperoxide-linked tissue damage. PBN is a nitrone-based free radical scavenger that reacts readily with many different types of free radicals forming nitroxide spin adducts which are generally much more stable and less reactive than the original free radicals [64]. At least theoretically, pretreatment with Ebselen or PBN would decrease the concentrations of free radicals released from PPC, PDO, or PN to activate TLR4, thereby inhibiting the induction of SEAP release. In the HEK-Blue null 1-v cells that do not express TLR4, oxidants did not induce SEAP release, which would indicate that SEAP release is specifically due to the stimulation of TLR4. Although Paul-Clark et al [65] reported no direct involvement of TLR4 with oxidant stimulation in HEK-293 cells transfected with TLR4, but our data failed to support this aspect of their findings. The discrepancy between the conclusions reached by Paul-Clark’s group and the present study could be due to the type of prooxidants used and the end-points in both studies. They used cigarette smoke condensate as a source of oxidants (*versus* PPC, PDO, or PN) with the level of chemokine CXCL8 released as an end point (*versus* SEAP release through NFκB reporting system) in the present study. Furthermore, the plasmid encoding the TLR4, CD14, and MD-2 (3 components of the LPS receptor complex), as well as any reporter gene constructs inserted into HEK293T cells was not clearly defined.

Our present findings have also raised an important basic question. Why do antioxidants decrease the efficacy of MPLA (a specific agonist for TLR4) in inducing the release of SEAP from HEK-Blue mTLR4 cells? This question in effect implies that antioxidants can decrease the efficacy of NF-κB activation that could result in new cytokine/chemokine syntheses and release. We speculate that a partial answer may be due to an intrinsic oxidant/antioxidant component of TLR4. The ability of antioxidants to reduce MPLA-induced SEAP release suggests a common oxidant/antioxidant-dependent mechanism for TLR4 activation (Fig. 3, panel 3A) [66]. This would in effect decrease NF-κB activation, with its potential therapeutic implications. The intracellular redox status is vital for maintenance of cell homeostasis and for proper cell functions. Furthermore, cells commonly produce ROS/RNS as a part of the inflammatory process [3]. Many lines of evidence indicate that the redox status of the cell is involved in modulating NF-κB activation [67], [68], which provides a rationale for the effect of antioxidants on SEAP release in the present study.

Why does prooxidant activation of TLR4 differ from that of TLR2? Importantly, TLR2 and TLR4 share TIRAP adaptor protein, which is essential for the MyD88-dependent signaling pathway of both receptors [56]. This suggests that both receptors would be affected in relatively the same manner by the same ligands, but they are not. However, the mTLR2 gene construct lacks the MD2 co-receptor (present upstream in the mTLR4 gene construct), which is directly involved in ligand-binding and subsequent receptor activation. The absence of MD2 in TLR2 construct may lay the major difference in responses between the two receptors to prooxidant effects. In the present study, among the three different agents used to produce oxidative species, PPC was the only one that induced significant SEAP release through TLR2 stimulation, and at five times higher concentration than was used to stimulate TLR4 in a robust manner. In addition, despite the higher concentration of PPC, the amplitude of PPC-induced activation of TLR2 was much lower than that quantified for TLR4 stimulation by PPC. Only LPS-PG (a specific activator of TLR2), but not MPLA (a specific activator of TLR4) induced SEAP release through TLR2 activation, which confirmed the specificity of LPS-PG and MPLA towards TLR2 and TLR4, respectively. It is most likely that the use of MPLA to activate TLR4 instead of the biologically standardized LPS used in many studies may have contributed to a differential specificity of TLR4 from TLR2 in the present study. It has previously been shown that a low level expression of TLR2 in cells can be up regulated through TLR4 signaling [69]. This suggests that a mechanism for inducible cross talk may exist between exogenous and endogenous stimuli. Indeed, exogenous ROS appear to cross talk with endogenous oxidases to activate NF-κB as demonstrated in an NADPH oxidase-deficient mouse model [70]. The magnitudes of the increases in NADPH levels were similar in response to either endogenous or exogenous oxidative stress [71]. Therefore, to clarify the role of TLR2 in exogenous oxidant signaling, it is essential to state that oxidants are potentially capable of activating TLR2 [72], [73], but at a much higher concentration than is needed to activate TLR4.

We have integrated our *in vitro* findings with an *in vivo* model of ONS associated with the induction of orofacial pain. To this end, we examined responses of mice to an orofacial inflammatory pain with IMM injection of PPC to mimic a model of “sterile” inflammation [74], [75]. PPC decays slowly *in vivo* to generate the same reactants that are produced physiologically by activated phagocytes [27], [37]. These reactants can maintain the inflammatory process for a long time by reducing cellular antioxidant defenses. PPC-induced inflammation has a clearly defined cause-effect links, unlike the adjuvant- and collagen-induced models in which immunogenic interferences quite frequently obscure their modes of action and make data interpretation difficult. The chromate (+VI) state, formed during the decay of PPC, appears to contribute to the progression of inflammation by the inhibition of glutathione reductase, thus increasing intracellular H_2_O_2_ concentrations. In addition, Cr (+VI) reduced to Cr (+V) by endogenous ascorbate, further catalyzes hydroxyl radical production in the presence of hydrogen peroxide. A stable loop would thus be formed, in which ROS, continuously produced by Cr (+VI)/Cr (+V) redox cycling, drives the primary response into chronic self-perpetuating inflammation. PPC-induced effects appear to be most relevant to a subset of human orofacial pain associated with metabolic arthritis [76]. Our *in vitro* data have shown the effect of pro-oxidants on TLR4 located on plasma membrane only. However, it is very likely that our *in vivo* immuno-blot determination of TLR4 included the expression of endosomal TLR4, which may also sense intracellular ROS/RNS.

We have used IL-1β and TNF-α as effective quantitative biomarkers of tissue activation (inflammatory response) as these cytokines are inducible by oxidants [77]. Masseter muscle inflammation appears to have peripheral, systemic and behavioral components. A peripheral component of orofacial pain would include peripheral production of proinflammatory cytokines such as TNF-α and IL-1β in the inflamed tissue and the spinal trigeminal complex [78]. The cytokines potentially derived from activated macrophages and other cell types including astrocytes and myocytes, can sensitize masseter muscles in the absence of gross inflammation. The masseter muscle ipsilateral to the PPC treatment showed not only increased response to mechanical stimulation quantified as MHWT, but also enhanced the expression of the proinflammatory cytokines, TNFα and IL-1β. On the other hand, the contralateral masseter “remote” from the site of PPC injection *vis-à-vis* source of oxidative stress did not exhibit significant changes in pain response or increased tissue levels of proinflammatory cytokines.

The kinetics of direct NF-κB activation by oxidants still remains not fully understood. However, we speculate that the prooxidants produced by PPC are responsible for both changes in orofacial pain sensitivity and cytokine levels in the ipsilateral masseter. Our conclusion is given more credence as EUK-134, a serum-stable scavenger of oxidative species [29], significantly decreased both tissue levels of cytokines and attenuated the pain response. In accord with a human orofacial pain syndrome such as the TMD [79], [80], we have shown increased masseter levels of TNF-α and IL-1β, which are two downstream proinflammatory cytokines of the TLR4 signaling pathway. A behavioral component of orofacial pain includes a decreased mechanical threshold for head withdrawal from a stimulus [81], which involves an intact trigeminal pain pathway. Mechanical hyperalgesia is of clinical relevance in many pain states. Exposure to exogenous oxidants may prime responsive cells for extended response to endogenous oxidants [82], [83]. This observation supports our hypothesis that exogenous oxidants can sensitize cells to initiate, propagate and maintain inflammation-induced ONS that may lead to chronic inflammatory pain/disease state.

In conclusion, we have shown that exogenous oxidants can activate cells via mainly through a TLR4-dependent pathway, with TLR2 potentially acting in synergy with TLR4 at a higher prooxidant concentration. Taken together, our findings suggest that ROS/RNS cycle through the TLR4 and its signaling pathway may be associated with initiation, propagation and maintenance of “sterile” inflammatory pain. We have also shown a relationship between oxidant- and PAMP−/DAMP-induced NF-κB activation through TLR4 stimulation. Our results further suggest that antioxidants may serve as useful adjuvants in the management of orofacial pain in combination with a modulator(s) of TLR4 signaling pathway [84], [85]. Therefore, TLR4 may thus represent a potential new therapeutic target for pain of different etiologies [86].
